# Pathophysiological Mechanism of Neurodevelopmental Disorders—Overview

**DOI:** 10.3390/cells11244082

**Published:** 2022-12-16

**Authors:** Koh-ichi Nagata

**Affiliations:** Department of Molecular Neurobiology, Institute for Developmental Research, Aichi Developmental Disability Center, 713-8 Kamiya, Kasugai 480-0392, Japan; knagata@inst-hsc.jp

Technological advancements in next-generation DNA sequencing have enabled elucidation of many genetic causes of neurodevelopmental disorders (NDDs) over the last two decades. However, the investigation of the molecular mechanisms underlying the pathophysiology of NDDs has advanced far less quickly, because multiple interdisciplinary experiments, which are generally low throughput, are essential for this purpose. This Special Issue of *Cells* is devoted to the recent advances in the identification of the molecular basis of NDDs, as well as the pathophysiological basis of these disorders. We invited original research articles and reviews on the “Pathophysiological Mechanisms of Neurodevelopmental Disorders”. Compiling articles that explore these mechanisms from molecular to cellular, tissue and animal model perspectives, as well as potential therapeutic strategies, this Special Issue successfully features various achievements in this rapidly expanding field by collating new knowledge and presents the state of the art for a wide range of NDDs.

The organized structure and spatial organization of the human brain is regulated by highly coordinated developmental events, such as neural cell proliferation, migration and differentiation. Sapir et al. employed a developmental perspective to describe and understand the etiology of common cortical malformations and their manifestation in the human brain [1].

As for the organella function, the endosomal recycling is executed by the two protein complexes, Retromer and Retriever, whose defects cause dysregulation of many membrane proteins and result in several disorders, including NDDs. Saitoh reviewed the biological and developmental roles of the two complexes, and the consequences of defects in endosomal recycling [2]. 

Rho family small GTPases regulate cellular signaling and cytoskeletal dynamics, playing a pivotal role in cell adhesion, migration, and cell-cycle progression. Among the Rac subfamily of Rho GTPases, the proper functions of Rac1 and Rac3 are crucial for neural development, and pathogenic variants affecting their biological processes are implicated in NDDs. Scala et al. reviewed the current pertinent literature on Rac-related disorders with a primary neurological involvement and provided an overview of the current knowledge on the pathophysiological mechanisms involved in the neuro-RACopathies [3].

In addition to a direct deleterious effect produced by genetic variants in the *RAC1/3* genes, dysregulated RAC1/3 functions resulting from pathogenic variations of genes encoding guanine nucleotide-exchange factors (GEFs) and GTPase-activating proteins (GAPs) have been involved in the pathogenesis of NDDs. A RhoGAP, Oligophrenin-1 (OPHN1), is enriched at the synapse and plays a critical role in cytoskeleton remodeling and vesicle recycling. The *OPHN1* gene mutations are associated with X-linked ID (XLID). Cresto et al. clarified that OPHN1 function in synapses is differentially affected during maturation of the brain, which provides some opportunities for early therapeutic intervention [4].

*CNKSR2*, which encodes a scaffolding molecule CNKSR2, is also responsible for XLID. CNKSR2 interacts with different molecules and regulates the MAP kinase (mitogen-activated protein kinase) cascade and Rho family small GTPase signaling. Ito and Nagata reviewed the possible pathophysiological mechanism of XLID by disrupting CNKSR2 functions, and summarized molecular features, neuronal function and NDD-related variations of *CNKSR2* [5]. 

Protein phosphorylation plays critical roles in a variety of intracellular signaling pathways in the brain, of which dysregulation has been implicated in NDDs. Ahammad et al. reported a novel online database to provide information about the phosphorylation signals identified using the “KANPHOS” (Kinase-Associated Neural Phospho-Signaling) database [6]. The authors obtained phosphoproteomics data for receptor-mediated MAP-kinase signaling in the striatum/nucleus accumbens, registered them in KANPHOS, and analyzed the related pathways.

Williams syndrome (WS) is a multisystem NDD caused by a de novo hemizygous deletion of ~26 genes from chromosome 7q11.23, including the transcription factor II-I (*GTF2I*). By studying a novel murine model with selective deletion of *Gtf2i* in the excitatory neurons of the forebrain, Grad et al. demonstrated the substantial effect a single gene deletion can exert on miRNA regulation and brain structure and advanced our understanding of WS [7].

Although circadian rhythms are essential for maintaining body health, the effects of chronic circadian disruption during neurodevelopment remain elusive. Using a mouse model, Fang et al. investigated the molecular and behavioral changes after circadian disruption [8]. They demonstrated molecular, cellular and behavioral changes in the model mice, and highlighted the critical role of circadian rhythms in neurodevelopment.

Congenital disorders of glycosylation (CDG)—inherited metabolic diseases caused by defects in glycosylation—are associated with ID. Okamoto et al. examined two siblings with ID, dysmorphic features, early onset epileptic encephalopathy, cerebral infarction, etc. [9]. Whole-exome sequencing clarified that both patients were homozygous for a novel pathogenic variant of *MAN1B1* (NM_016219.4:c.1837del) inherited from their healthy parents. While subsequent HPLC analysis clarified N-glycan-processing defects, MAN1B1 activity was compromised in patient-derived lymphocytes.

Seizure threshold 2 (SZT2) is a component of the KICSTOR complex which regulates the mechanistic target of Rapamycin complex 1 (mTORC1). Mutations in this gene cause NDDs. Cattelani et al. performed a systematic interactome analysis of SZT2 and identified clusters of proteins related to autophagy, ciliogenesis regulation, neurogenesis and neurodegenerative processes [10]. They then investigated the physiological functions of SZT2 that could explain major molecular events in the pathophysiology of developmental and epileptic encephalopathy in patients with SZT2 mutations.

Bertacchi et al. focused on the pathophysiology of Bosch–Boonstra–Schaaf Optic Atrophy Syndrome (BBSOAS), a recently described monogenic neurodevelopmental syndrome caused by the haploinsufficiency of *NR2F1* gene, encoding a transcriptional regulator [11]. The authors highlighted what is missing to achieve a full comprehension of BBSOAS pathogenesis and proposed future experimental paths that could translate discoveries from the bench to the clinic.

AUTS2 is involved in transcriptional regulation, neuronal migration and neuritogenesis during brain development, while AUTS2 postnatally regulates the number of excitatory synapses to maintain the balance between excitation and inhibition in neural circuits. Notably, *AUTS2* has emerged as a crucial gene associated with a wide range of NDDs. Hori et al. summarize the knowledge regarding AUTS2, including its molecular and cellular functions in neurodevelopment, its genetics and its role in behaviors [12].

Deficits in the inhibitory circuits by interneurons that regulate the excitatory–inhibitory (E–I) balance toward excitation have been implicated in NDDs, including Fragile X syndrome (FXS). Indeed, manipulating the interneuron activity ameliorated the symptoms in the FXS mouse model, which makes it reasonable to conceptualize FXS as an interneuronopathy. While the ways in which the developmental profiles of the inhibitory circuit go awry in FXS are still poorly understood, recent works have uncovered several developmental alterations in the functional properties of interneurons. Nomura reviewed the recent evidence of the inhibitory alterations and interneuron dysfunction in FXS and discussed the therapeutic potential of correcting disrupted E–I balance in FXS [13]. 

Chromosomal microarray testing reliably detects minute changes in genomic copy numbers. Haploinsufficiency is considered to be the pathogenic mechanism when the deletion of a gene is related to NDDs. While loss-of-function mutations may be evaluated using next-generation sequencing, increased copy numbers may exhibit different clinical symptoms; the additional copies of a gene may have a dominant negative effect. In addition, gene mutations with dominant negative effects are responsible for causing the clinical symptoms. Currently, the diagnostic yield of genomic alterations using comprehensive analysis is less than 50%, indicating the existence of more subtle alterations or genomic changes in the untranslated regions, while copy-neutral inversions and insertions may be related. Yamamoto outlined the genetic factors of NDDs and reviewed the current state of diagnosis by genomic medicine [14].

The Axon initial segment (AIS), proximally located at the interface between the axon and cell body, has characteristic molecular and structural properties, and is a key domain for the control of neuronal excitability by homeostatic mechanisms. The AIS has high plasticity in normal developmental processes and pathological activities including NDDs. Fujitani et al. provided an overview of the molecular and structural characteristics of AIS, as well as AIS regulation through axo-axonic synapses, and axo−glial interactions [15]. They then discuss the plasticity of AIS and the pathophysiological role of an abnormal AIS in NDDs.
